# Development and Application of an Interdisciplinary Rapid Message Testing Model for COVID-19 in North Carolina

**DOI:** 10.1177/00333549211018676

**Published:** 2021-05-19

**Authors:** Sophia M. Bartels, Katherine Gora Combs, Allison J. Lazard, Victoria Shelus, C. Hunter Davis, Allison Rothschild, Maura Drewry, Kathryn Carpenter, Emily Newman, Allison Goldblatt, Nabarun Dasgupta, Lauren M. Hill, Kurt M. Ribisl

**Affiliations:** 1 41474 Department of Health Behavior, Gillings School of Global Public Health, University of North Carolina at Chapel Hill, Chapel Hill, NC, USA; 2 2331 Carolina Population Center, University of North Carolina at Chapel Hill, Chapel Hill, NC, USA; 3 222941 Hussman School of Journalism and Media, University of North Carolina at Chapel Hill, Chapel Hill, NC, USA; 4 Lineberger Comprehensive Cancer Center, University of North Carolina at Chapel Hill, Chapel Hill, NC, USA; 5 Injury Prevention Research Center, University of North Carolina at Chapel Hill, Chapel Hill, NC, USA

**Keywords:** message testing, rapid design, COVID-19, social distancing, emergency preparedness

## Abstract

**Introduction:**

From the onset of the COVID-19 pandemic, public health officials have sought to develop evidence-based messages to reduce COVID-19 transmission by communicating key information to media outlets and the public. We describe the development of an interdisciplinary rapid message testing model to quickly create, test, and share messages with public health officials for use in health campaigns and policy briefings.

**Methods:**

An interdisciplinary research team from the University of North Carolina at Chapel Hill assembled in March 2020 to assist the state health department in developing evidence-based messages to influence social distancing behaviors in the state. We developed and iteratively executed a rapid message testing model; the components of the 4-step model were message creation, survey development, survey administration, and analysis and presentation to health department officials. The model was executed 4 times, each during a 7-day period in April and May, and each subsequent survey included new phrasing and/or messaging informed by the previous week’s survey. A total of 917 adults from North Carolina participated in the 4 surveys.

**Results:**

Survey participants rated messages focused on protecting oneself and others higher than messages focused on norms and fear-based approaches. Pairing behaviors with motivations increased participants’ desire to social distance across all themes and subgroups. For example, adding “Protect your grandmother, your neighbor with cancer, and your best friend with asthma,” to messaging received a 0.9-point higher score than the base message, “Stay 6 feet apart from others when out in public.”

**Practice Implications:**

Our model to promote social distancing in North Carolina during the COVID-19 pandemic can be used for rapid, iterative message testing during public health emergencies.

The number of COVID-19 cases, hospitalizations, and deaths increased steadily in North Carolina in 2020.^1^ In July 2020, North Carolina was designated a “red zone” state, that is, a state with a rate of >100 new cases per 100 000 people and a test positivity rate >10%.^2^ The highest incidence of COVID-19 cases in North Carolina centered on its largest cities and disproportionately affected many racial and ethnic minority groups.^1^

Social distancing is 1 of the 3 pillars of North Carolina’s strategy for decreasing the number of cases of COVID-19.^3^ Social distancing includes keeping a distance of at least 6 feet from people not from one’s household, among other suggestions.^3,4^ Infectious disease modeling of COVID-19 demonstrates the necessity of social distancing to reduce the incidence of disease and prevent the health care system from being overwhelmed.^5^ The Centers for Disease Control and Prevention (CDC) lists social distancing as the most effective way to reduce the spread of COVID-19, because the route of COVID-19 transmission is mainly among people who are within 6 feet of each other for an extended period.^4^

At the start of the COVID-19 pandemic, the need for evidence-based messaging for preventive behaviors, especially social distancing and staying home when possible, among North Carolina residents was clear. Because of the rapidly escalating number of cases, these messages also needed to be expedited. Building partnerships with state health departments and university research teams is one way to accelerate the translation of messaging research to practice. We describe a rapid message testing model centered on a partnership between the North Carolina Department of Health and Human Services (NCDHHS) and the University of North Carolina (UNC) at Chapel Hill. This model allowed our team to develop messages with a broad impact in North Carolina (ie, common denominator communication approach) and identify whether messages resonated among certain groups (eg, rural populations).^6,7^ Our approach could be applied in other states to develop messaging to prevent the spread of COVID-19 and other emerging threats.

An interdisciplinary research team from UNC with expertise in health communication, epidemiology, health behavior, and public policy quickly assembled to address NCDHHS’s needs. The interdisciplinary nature of this team was vital because it allowed us to develop messages in real time that were informed by the combined expertise and theories of the 20 or so team members. We aimed to develop messages that resonated with all North Carolina residents, including several key populations: rural residents, populations that are disproportionately affected by COVID-19 (eg, Black/African American people), and people perceived as less likely to social distance (eg, young adults and Republicans).

Although numerous messaging processes exist,^8-10^ they do not incorporate perspectives from multiple disciplines in real time, are not agile enough to rapidly develop and test messages in emergency situations, or are unable to respond quickly to changing environments and health department needs. Other messaging approaches have incorporated one of these elements, but our approach is uniquely applicable to the context of emergency situations. We describe the creation and implementation of an interdisciplinary rapid message testing model, which was implemented during the course of 4 surveys, in the context of the COVID-19 pandemic and how it could be used to guide research teams in their work with health departments to develop messages during a public health emergency.

## Materials and Methods

### Research Team

An interdisciplinary team of undergraduate and graduate research assistants, faculty, and staff members from UNC assembled in March 2020 to assist NCDHHS in developing evidence-based messages to influence social distancing behaviors in North Carolina. Team members were from the UNC Hussman School of Journalism and Media, the Gillings School of Global Public Health, and the Department of Political Science. The team of volunteers quickly assembled without institutional constraints (eg, hiring freezes) during the rapidly escalating pandemic. Our interdisciplinary team approached the social distancing messaging from various perspectives. For example, communication experts provided guidance on strategic message development, and health behavior experts contributed their understanding of health behavior change. Team members from epidemiology provided insight on public health–informed guidelines for social distancing behaviors, and political science experts provided evidence for population-based values and sociodemographic nuances predictive of social distancing.

The research team was split into workgroups for tasks associated with each iterative step of the rapid message testing model—a common process for health communication development.^11^ Each workgroup consisted of enough members to allow flexible scheduling and substitution if someone was unavailable (eg, if 2 members were essential, then the group had 3 or 4 members). At each step, faculty in the research group mentored the research assistants. The UNC Institutional Review Board deemed this research as exempt.

### Rapid Message Testing Model

Our rapid message testing model was iteratively executed during a 7-day period using an expedited common health communication development process.^11^ Step 1 was message creation. On days 1 and 2, two research assistants developed messages to reach all residents in North Carolina. Messages were developed based on recommended behaviors from NCDHHS and public health faculty, social distancing messages from other states, and key theoretical predictors for health behavior change (eg, attitudes, risk perceptions).^12-17^ For example, people may want to socially distance because they want to protect themselves or people at high risk of contracting COVID-19 (perceived severity), feel pressure from family (social norms), or are able and feel a responsibility to do so (self-efficacy). We chose to focus on motivations, or reasons for social distancing, rather than barriers to compliance, because barriers were not well-studied or understood in North Carolina initially. Assessing how much a message motivates—or discourages—action is strongly correlated with actual behavior in other health contexts and is a promising approach for novel health behavior decisions.^18^

Step 2 in our rapid message testing model was the development of the week’s social distancing message evaluation survey, which tested phrases or messages to encourage social distancing among North Carolina residents. One research assistant used the evaluation goals to create a codebook with programming directions and measures, and a second research assistant programmed the codebook in Qualtrics (Qualtrics Intl), an online survey software. The codebook included new messages from the message development team, items to evaluate these messages, and sociodemographic questions. The codebook was approved by the leadership team before being programmed. Once programmed, the codebook developer and a member of the leadership team tested and finalized the codebook. The codebook was created on days 3 and 4 and programmed and tested on the evening of day 4.

Step 3 was administering the survey to North Carolina residents aged ≥18. On day 5, the survey went live on Amazon Mechanical Turk (MTurk), a crowdsourcing platform often used for communication and social science research^19,20^ and ran until the morning of day 6, or until we had reached about 200 participants. Using MTurk—an established, accessible survey platform—allowed our team to quickly reach participants remotely, given that in-person message testing was not viable because of the pandemic, and filter for participants who lived in North Carolina. We selected a sample size of about 200 participants because of the feasibility of recruitment within our rapid time frame and to have a large enough sample to enhance our ability to detect within-subject differences.^21^ A total of 917 adults from North Carolina participated in the 4 surveys. Participants were aged 18-72, with a mean (SD) age of 41 (12.8) in survey 1, 37 (12.9) in survey 2, 37 (12.5) in survey 3, and 37 (13.0) in survey 4. Most participants were White, female, and from suburban areas (Table 1). All participants provided consent electronically before participating in each survey.

**Table 1:** Characteristics of participants aged ≥18 in 4 surveys used to assess the salience of social distancing messages during the COVID-19 pandemic, North Carolina, April 9–May 7, 2020^a^

Characteristic	Survey 1 (n = 267)	Survey 2 (n = 250)	Survey 3 (n = 200)	Survey 4 (n = 200)	North Carolina population, %^b^
Age, mean (SD) [range], y	41 (12.8) [19-72]	37 (12.9) [18-71]	37 (12.5) [18-69]	37 (13.0) [18-72]	—
Sex					
Male	118 (44.4)	108 (43.2)	92 (46.0)	93 (46.5)	48.7
Female	145 (54.5)	142 (56.8)	107 (53.5)	107 (53.5)	51.3
Other	3 (1.1)	0	1 (0.5)	0	
Race^c^					
White	217 (81.3)	199 (79.6)	150 (75.0)	151 (75.5)	70.9
Black or African American	38 (14.2)	31 (12.4)	29 (14.5)	31 (15.5)	23.0
American Indian/Alaska Native	6 (2.2)	5 (2.0)	9 (4.5)	8 (4.0)	2.0
Asian	12 (4.5)	18 (7.2)	16 (8.0)	14 (7.0)	3.5
Native Hawaiian/Other Pacific Islander	0	2 (0.8)	1 (0.5)	2 (1.0)	0.2
Other	1 (0.4)	3 (1.2)	2 (1.0)	3 (1.5)	3.4
Ethnicity^d^					
Hispanic/Latino	9 (3.4)	13 (5.2)	14 (7.0)	12 (6.0)	9.4
Not Hispanic/Latino	257 (96.3)	237 (94.8)	184 (92.0)	187 (93.5)	90.6
Education					
<High school graduate	1 (0.4)	2 (0.8)	2 (1.0)	0	—
High school graduate/GED	35 (13.1)	15 (6.0)	14 (7.0)	20 (10.0)	—
Some college or technical school	48 (18.0)	54 (21.6)	35 (17.5)	35 (17.5)	—
Associate’s degree	34 (12.7)	37 (14.8)	31 (15.5)	27 (13.5)	—
Bachelor’s degree	111 (41.6)	103 (41.2)	84 (42.0)	88 (44.0)	—
Graduate or professional degree	38 (14.2)	39 (15.6)	34 (17.0)	30 (15.0)	—
Political identity^e^					
Democrat/lean Democrat	129 (48.3)	127 (50.8)	100 (50.0)	90 (45.0)	—
Independent/no lean	51 (19.1)	58 (23.2)	41 (20.5)	43 (21.5)	—
Republican/lean Republican	87 (32.6)	65 (26.0)	59 (29.5)	67 (33.5)	—
Rurality					
Urban	64 (24.0)	59 (23.6)	47 (23.6)	53 (36.5)	—
Suburban	123 (46.1)	119 (47.6)	103 (51.8)	99 (49.5)	—
Rural	80 (30.0)	72 (28.8)	49 (24.6)	48 (24.0)	—

Abbreviation: GED, general education development.

^a^Missing data for several questions explain the difference between the number of responses for each variable and the total number of respondents for each survey. All values are number (percentage) unless otherwise indicated.

^b^North Carolina population information is from the American Community Survey 5-year estimates for 2019.^22^

^c^Race alone or in combination with ≥1 other race.

^d^Of any race.

^e^Democrat/lean Democrat includes: Democrat, strongly; Democrat, not so strongly; Independent, but lean toward Democrat. No lean includes: Independent. Republican/lean Republican includes: Independent, but lean toward Republican; Republican, not so strongly; Republican, strongly.

Step 4 was analyzing survey results, interpreting findings, and presenting insights to key members of NCDHHS. A research assistant analyzed the survey results on the evening of day 6, using SPSS version 26 (IBM Corp). Key findings, summary descriptive statistics (eg, means), message rankings, and differences in message ranking by subgroup breakdowns were shared for interpretation and development of the week’s message framework. In survey 4, we also examined differences in message theme—comparing a baseline behavioral message with other messages with an added motivation—using repeated-measures analyses of variance. Results were packaged succinctly to be directly usable by NCDHHS. On day 7, project leadership shared findings and suggested message strategies. In this meeting, the team heard from NCDHHS how priorities had or had not changed and received guidance for selecting messages for further testing. After the meeting, the entire team discussed findings, incorporated new guidelines, and strategized next steps. This feedback then restarted the rapid testing cycle.

Messaging strategies shared each week with government officials were directly and immediately incorporated into public COVID-19 briefings for North Carolina residents and informed messages delivered throughout the state on retail signage and social media.

### Iterative Survey Development

Each anonymous weekly survey executed during the rapid message testing model included items for new phrasing and/or messages. Surveys 1-4 were launched on April 9, 15, and 22 and on May 6, 2020, respectively. Insights from each survey informed subsequent surveys in this interdependent process. In survey 1, we focused on phrase salience and understanding motivations. Participants rated how much reasons for social distancing made them want to comply with recommendations and preferred phrasing, on a scale ranging from 1 (not at all) to 5 (a great deal). In survey 2, we focused messaging on 4 behaviors: (1) minimizing in-person socializing with people outside the household, (2) not having anyone from outside the household in one’s home, (3) leaving one’s home only for essential items and exercise, and (4) staying at least 6 feet away from others when leaving one’s home. The key motivations and phrases from survey 1 were incorporated into these messages. In survey 3, we developed 5 themes from the high-scoring messages from survey 2: (1) save lives, (2) protect people you love, (3) keep your loved ones safe, (4) a personalized promise (“For my [friend or family member]—I promise to stay inside for you”), and (5) change is tough. Theme 5 was added based on the literature and on team member recommendations showing the importance of challenging a resistance-to-change mindset.^23^ In survey 4, we retained well-performing themes and systematically paired enduring social distancing behaviors with motivations. As the pandemic response was evolving, we shifted our focus from messages centered on not leaving one’s home, based on concerns of stigmatizing people who were unable to stay home (ie, essential workers). In addition, new CDC recommendations encouraged wearing face coverings in public, and focus shifted to staying home if sick rather than staying home in general,^24^ as states began to ease restrictions.

## Results

In survey 1 (content identification), participants were most motivated to keep others (eg, family members, people at high risk for contracting COVID-19) or themselves safe and were not motivated by protecting their image (eg, not wanting to be blamed for spreading the virus). Results were similar among subgroups. High-scoring motivations and phrases from survey 1 were incorporated into the messages tested in our second survey. From survey 2 (message refinement), we learned that people in North Carolina preferred general messages (eg, stay home to save lives) rather than specific suggestions (eg, not socializing in your driveway). They also preferred general messages rather than highlighting specific behaviors as “valid” or “invalid” (eg, “making a trip to the store just to get your favorite ice cream is an invalid reason to leave home”). Residents also responded well to messages that provided examples of people they are protecting by social distancing.

Using these results, we identified 5 themes, noted in the Methods section, to refine in survey 3. From survey 3 (message refinement), our data further demonstrated that a focus on motivations (eg, “stay home to protect your grandmother”) was important to encourage social distancing. We also found that people did not like messages that focused on a change mindset (eg, “Change is tough”). Finally, from survey 4, we found that pairing behaviors with motivations substantially increased desires to social distance across all themes and subgroups. For example, adding “Protect your grandmother, your neighbor with cancer, and your best friend with asthma,” on average had a 0.9-point higher score than the base message, “Stay 6 feet apart from others when out in public,” a substantial increase (Table 2).

**Table 2:** Rankings of example social distancing survey messages from 4 surveys, by key population group, North Carolina, April 9–May 7, 2020^a,b^

Message example	All study participants		Adults aged 18-24		Black or African American		Republican		Rural	
	Mean	Rank	Mean	Rank	Mean	Rank	Mean	Rank	Mean	Rank
**Survey 1**										
I want to protect people who are vulnerable.	4.5	1/19	4.4	4/19	4.6	2/19	4.5	2/19	4.5	3/19
I feel pressure from friends and family.	2.2	17/19	3.1	16/19	2.5	17/19	2.1	17/19	2.0	17/19
**Survey 2**										
You can make a difference. Stay home to protect your grandmother, your neighbor with cancer, and your best friend with asthma.	4.3	1/11	4.2	3/11	4.3	2/11	4.3	1/11	4.2	3/11
Irresponsible socializing is having a conversation with your neighbors in your driveway or grocery store.	2.8	11/11	3.0	11/11	3.3	11/11	2.8	11/11	2.7	11/11
**Survey 3**										
Older adults are at higher risk for severe illness from COVID-19. You can make a difference. Stay home to save lives.	4.2	1/9	4.1	1/9	4.4	1/9	4.2	2/9	4.0	4/9
Change is tough, but here is your chance to protect your community. Stay home to save lives.	3.7	5/5	3.6	4/5	3.8	5/5	3.7	5/5	3.4	5/5
**Survey 4**										
Protect your grandmother, your neighbor with cancer, and your best friend with asthma.	4.0	1/8	4.3	1/8	4.3	2/8	4.2	1/8	3.9	2/8
Stay 6 feet apart from others when out in public.	3.2	8/8	2.8	8/8	3.9	8/8	3.2	8/8	3.0	8/8

^a^Messages fell within categories or themes (eg, category: “reasons for social distancing;” theme: “socializing with people [in-person] outside of your household should be minimized”). Messages were ranked based on mean score within a theme or category; the denominator for “rank” is the number of messages within each theme or category.

^b^Messages were scored on a Likert-type scale (1 = not at all, 2 = a little, 3 = somewhat, 4 = quite a bit, 5 = a great deal).

## Discussion

One major barrier to evidence-based health communication and policy making is the time it takes for new evidence to be disseminated and used. Although it can take years from research question development to dissemination of findings, in emergency situations policy makers need to quickly know the most promising messages. In addition, the work of policy makers and researchers is often done in isolation, leading to evidence that is not useful to the policy maker and a lack of evidence-based policies.^25-27^

Our interdisciplinary model for rapid message testing, in this implementation context, reduces the time needed for evidence-based messages to reach policy makers. This approach increases the relevance of research for policy makers and public health officials. By basing message development on feedback from state agencies, researchers can respond to policy makers’ changing needs on a weekly basis, a necessity in public health crises such as the COVID-19 pandemic. This approach draws on elements of lean and agile principles, which are centered on eliminating waste to reach an “ideal process” and incorporating knowledge into practice through rapidly developing usable prototypes based on testing, user feedback, and validation.^28,29^ Agile designs have been used in other areas of COVID-19 research, such as the development of applications to improve well-being during social distancing.^30^ As the threat of global disasters and pandemics increases with climate change and population growth, these innovative models fill the need for evidence-based messages, products, and interventions that are developed quickly and respond to changing conditions.^31,32^

The rapid message testing model has 4 strengths. The first 2 strengths are its speed and responsiveness. The model allows evidence-based messaging to be quickly available to policy makers and can be used by public health departments to communicate with the public about health behaviors. The model’s ability to be iterative encourages stronger messages with each survey and allows for changes to be made to the messages with the discovery of new information. We found that general messages to protect others and oneself were the most motivating across all participants and subgroups, whereas fear-based messages, which other research has found to be ineffective in promoting behavior change, were not motivating.^33^ This model would also allow teams to detect whether messages resonate differently among subgroups for targeted communication (eg, unique messages could be designed for people at various levels of readiness to adopt behaviors). The third strength is that an interdisciplinary team created the model. By including faculty, staff members, and students from multiple departments, message development and evaluation benefited from the combined strength of theories and methodologies. Public health challenges, such as global pandemics, that are multidimensional and complex can be addressed only through the merging of multiple perspectives and disciplines.^34^ The final strength of the model is its cost-effectiveness. Leveraging accessible consumer panels (eg, MTurk) and working in real-time collaborations reduces overhead associated with traditional research methods. As opposed to studies that require substantial investment with no guarantee that the results will reach policy makers or be implemented, our model is designed to provide public health officials with evidence-based messages.^35^

This model also has several limitations. First, rapidly changing knowledge about COVID-19 transmission resulted in modifications to the key behaviors across each survey (ie, from social distancing only to social distancing and wearing face coverings), similar to campaign strategies that have shifted with emerging evidence for how to best prevent infection.^36-38^ This change in key behaviors required alterations to previously successful messages, which then had to be retested and iterated with input from NCDHHS. However, this limitation was specific to the volatility of the pandemic and would likely be less of a problem for better-understood health behaviors and disasters where messaging can be tracked during longer periods.^39^ Second, the rapid time frame of this model, along with the use of a crowdsourcing platform, made it difficult to achieve a representative sample in each survey. Specifically, systematic barriers, such as internet connectivity, reduce the ability of platforms, such as MTurk, to reach diverse populations. In addition, the platform does not allow for the recruitment of a probabilistic sample. These characteristics limit the generalizability of findings to the target audience. To improve targeting of messages for groups at high risk of contracting COVID-19, additional data collection methods, such as participatory research techniques, should be considered.^26^ Third, the suggested time frame can make it difficult to generate new messages each week while also amending messaging in response to changing knowledge about COVID-19. This challenge can be addressed by ensuring an appropriate-sized team (which may vary with the scope of the project) with the ability to produce the expected deliverables, although quickly forming an interdisciplinary team that is not voluntary may introduce additional challenges. We recommend having enough team members to rotate through tasks to allow for roles to be covered if someone is not available. Finally, our team used a single item to assess the perceived effectiveness of the message, whereas a multi-item scale may have produced scores that better incorporated all dimensions of message salience.

## Practice Implications

The proposed rapid message testing model provides a process for researchers to place evidence-based messages in the hands of public health departments and policy makers in real time. This model may be especially helpful during times of national disasters and public health crises. Further research should examine the feasibility of this model for other public health challenges outside of COVID-19. Research should also determine if the utility of this model holds with a range of health areas and if replicated across states. Researchers should also examine if the inclusion of qualitative and participatory research techniques increases positive reception and reach of the messages produced.^40^
